# Supplementary material for: Mechanisms governing the pioneering and redistribution capabilities of the non-classical pioneer PU.1
Source: Nat Commun. 2020 Jan 21;11:402. doi: 10.1038/s41467-019-13960-2 (PMC6972792; doi:10.1038/s41467-019-13960-2)
Supplement: Supplementary file 7 — Source data [file 41467_2019_13960_MOESM7_ESM.zip › Source_Data/FigureS2/FigureS2B_MotifScanOutput/KmeansCluster11/homerResults/motif18.similar.html]

motif18

## Information for motif18

A
T
G
C
G
C
T
A
T
A
G
C
A
C
G
T
A
C
G
T
A
G
T
C
A
C
G
T
A
T
C
G
A
G
T
C
G
C
A
T
G
C
A
T
  
Reverse Opposite:  

C
G
T
A
C
G
T
A
C
T
A
G
A
T
G
C
C
G
T
A
A
C
T
G
C
G
T
A
C
G
T
A
A
T
C
G
C
G
A
T
T
A
C
G
  

|  |  |
| --- | --- |
| p-value: | 1e-25 |
| log p-value: | -5.971e+01 |
| Information Content per bp: | 1.747 |
| Number of Target Sequences with motif | 127.0 |
| Percentage of Target Sequences with motif | 3.91% |
| Number of Background Sequences with motif | 542.1 |
| Percentage of Background Sequences with motif | 1.20% |
| Average Position of motif in Targets | 103.2 +/- 40.4bp |
| Average Position of motif in Background | 101.8 +/- 67.3bp |
| Strand Bias (log2 ratio + to - strand density) | -0.3 |
| Multiplicity (# of sites on avg that occur together) | 1.02 |
| Motif File: | file (matrix) reverse opposite |

### Similar de novo motifs found

|  |  |  |  |  |  |  |  |
| --- | --- | --- | --- | --- | --- | --- | --- |
| Rank | Match Score | Redundant Motif | P-value | log P-value | % of Targets | % of Background | Motif file |
| 1 | 0.908 | T A C G C T G A C T G A C T G A C T A G A T G C C G T A A T C G C G T A C T G A A C T G C A G T A T C G T A G C | 1e-22 | -50.819416 | 0.95% | 0.05% | motif file (matrix) |
| 2 | 0.948 | A G T C C G T A T A G C C G A T A C G T T G A C C G A T A T C G A G T C G C A T | 1e-21 | -49.059602 | 3.60% | 1.20% | motif file (matrix) |
| 3 | 0.958 | A G T C C G T A A G T C A C G T A C G T A G T C A C G T A T C G A G T C | 1e-18 | -42.952132 | 4.37% | 1.79% | motif file (matrix) |
| 4 | 0.938 | A G T C C G T A A G T C A C G T A C G T A G T C A C G T A T C G | 1e-16 | -37.596062 | 5.69% | 2.82% | motif file (matrix) |
